# Surgical management of chronic Achilles tendon rupture: evidence-based guidelines

**DOI:** 10.1186/s13018-024-04559-5

**Published:** 2024-02-10

**Authors:** Shi-Ming Feng, Nicola Maffulli, Francesco Oliva, Amol Saxena, Yue-Feng Hao, Ying-Hui Hua, Hai-Lin Xu, Xu Tao, Wei Xu, Filippo Migliorini, Chao Ma

**Affiliations:** 1 Orthopaedic Department, Sports Medicine Department, Xuzhou Central Hospital, No. 199, the Jiefang South Road, Xuzhou, 221009 Jiangsu China; 2 Department of Medicine and Psychology, University “La Sapienza”, Rome, Italy; 3 Guy Hilton Research Centre, School of Pharmacy and Bioengineering, Keele University, Stoke-On-Trent, Staffordshire, ST4 7QB England; 4 Centre for Sports and Exercise Medicine, Barts and The London School of Medicine and Dentistry, Mile End Hospital, 275 Bancroft Road, London, E1 4DG England; 5 Department of Sports Traumatology, Universita’ Telematica San Raffaele, Rome, Italy; 6 Department of Sports Medicine, Sutter-PAMF, Palo Alto, CA USA; 7 Orthopedics and Sports Medicine Center, Suzhou Municipal Hospital, Nanjing Medical University Affiliated Suzhou Hospital, Suzhou, Jiangsu People’s Republic of China; 8 Department of Sports Medicine, Huashan Hospital, Fudan University, Shanghai, People’s Republic of China; 9 Department of Trauma and Orthopedic, People’s Hospital, Peking University, Beijing, People’s Republic of China; 10 Department of Sports Medicine, Southwest Hospital, Army Medical University, Chongqing, People’s Republic of China; 11 Department of Orthopaedics, The Second Affiliated Hospital of Soochow University, Suzhou, Jiangsu People’s Republic of China; 12 Department of Orthopaedic, Trauma, and Reconstructive Surgery, RWTH University Medical Centre, Pauwelsstraße 30, 52074 Aachen, Germany; 13 Department of Orthopedics and Trauma Surgery, Academic Hospital of Bolzano (SABES-ASDAA), Teaching Hospital of the Paracelsus Medical University, 39100 Bolzano, Italy

**Keywords:** Chronic Achilles tendon rupture, Surgical management, Guidelines

## Abstract

**Background:**

Chronic Achilles tendon ruptures (CATR) often require surgical intervention to restore function. Despite numerous treatment modalities available, the optimal management strategy remains controversial given the limited high-quality evidence available. This article aims to provide evidence-based guidelines for the surgical management of CATR through a comprehensive systematic review of the available data. The consensus reached by synthesizing the findings will assist clinicians in making informed decisions and improving patient outcomes.

**Methods:**

A group of 9 foot surgeons in three continents was consulted to gather their expertise on guidelines regarding the surgical management of CATR. Following the proposal of 9 clinical topics, a thorough and comprehensive search of relevant literature published since 1980 was conducted for each topic using electronic databases, including PubMed, MEDLINE, and Cochrane Library, to identify relevant studies published until 1 October 2023. All authors collaborated in drafting, discussing, and finalizing the recommendations and statements. The recommendations were then categorized into two grades: grade a (strong) and grade b (weak), following the GRADE (Grading of Recommendations Assessment, Development, and Evaluation) concept. Additionally, feedback from 21 external specialists, who were independent from the authors, was taken into account to further refine and finalize the clinical guidelines.

**Results:**

Nine statements and guidelines were completed regarding surgical indications, surgical strategies, and postoperative rehabilitation protocol.

**Conclusion:**

Based on the findings of the systematic review, this guideline provides recommendations for the surgical management of CATR. We are confident that this guideline will serve as a valuable resource for physicians when making decisions regarding the surgical treatment of patients with CATR.

## Introduction

The Achilles tendon is the largest and strongest tendon in the human body, connecting the muscles of the gastrosoleus complex to the calcaneus [1]. The Achilles tendon is important to maintain the standing posture, and during walking, running, and jumping [2, 3]. Tears of the Achilles tendon are classified as chronic after a four- or six-week period following the initial injury[4–10]. Patients with chronic Achilles tendon rupture (CATR) report weakness, instability, increased dorsiflexion of the ankle, swelling, tenderness, and thickening of the tendon, and, at times, persistent pain [11–17].

In the 1970s, surgical interventions for Achilles tendon ailments gained popularity as they offered better outcomes and lower re-rupture rates compared to non-surgical methods [18–20]. The traditional surgical technique involved an open repair, where the surgeon made an incision to directly access the ruptured tendon and suture it back together [21–23]. This approach showed good clinical and functional outcomes, but it often required a relatively large incision, and resulted in increased scar tissue formation[24, 25]. Minimally invasive [26–28] and percutaneous techniques have emerged as an alternative to open repair [29–33]. These techniques reduce surgical trauma, minimize scarring, and allow faster recovery. Advances in surgical techniques have further refined the management of CATR. The use of endoscopic or arthroscopic methods has gained popularity, enabling surgeons to visualize and repair the tendon through smaller incisions [34–37]. These minimally invasive procedures offer advantages such as reduced postoperative pain, faster recovery, and improved cosmetic outcomes [37–39]. Overall, the history of surgical management for CATR has witnessed a shift toward less invasive techniques, improved surgical outcomes, and the exploration of regenerative therapies [40, 41].

Currently, the treatment of CATR is highly challenging, with many techniques and marked variations in treatment methods [42–48]. There are also considerable differences among various surgical approaches, and consensus has not yet been reached [49–54]. Therefore, to further provide a reference for clinicians in selecting surgical methods for CATR, we have formulated this guideline through expert consultations and critical structured analysis of the published peer-reviewed literature. This guideline aims to comprehensively summarize the current research achievements and provide the most valuable information for the clinical practice of surgical treating CATR.

## Methods

The current guideline is intended for patients aged 16 and 60 years with CATR.

### Basic overview of the procedure

The process began in January 2023. Nine orthopedic surgeons were consulted to gather their expertise and insights on guidelines concerning the surgical management of CATR. A total of 9 topics were proposed, covering surgical indications, surgical strategies, and postoperative rehabilitation protocols. For each topic, comprehensive systematic searches were conducted in the literature published since 1980. Based on the findings, a draft of the guideline statement outlining the recommendations was prepared. The draft of the guideline statement was thoroughly reviewed, modified, and finalized by all the authors listed in this consensus statement. A consensus meeting was held in May 2023, when the evidence level and grading of the recommendations were determined. During the meeting, 21 specialist surgeons, all experienced in the treatment of CATR, engaged in discussions and modifications until a 70% agreement was achieved. These specialist surgeons were academic and non-academic orthopedics with at least 10 years of post-fellowship independent practice, of consultant status, who practiced in hospital where at least 5 chronic Achilles tendon ruptures per year were managed (range 5–18).

### Method of systematic literature review

**Fig. 1:**
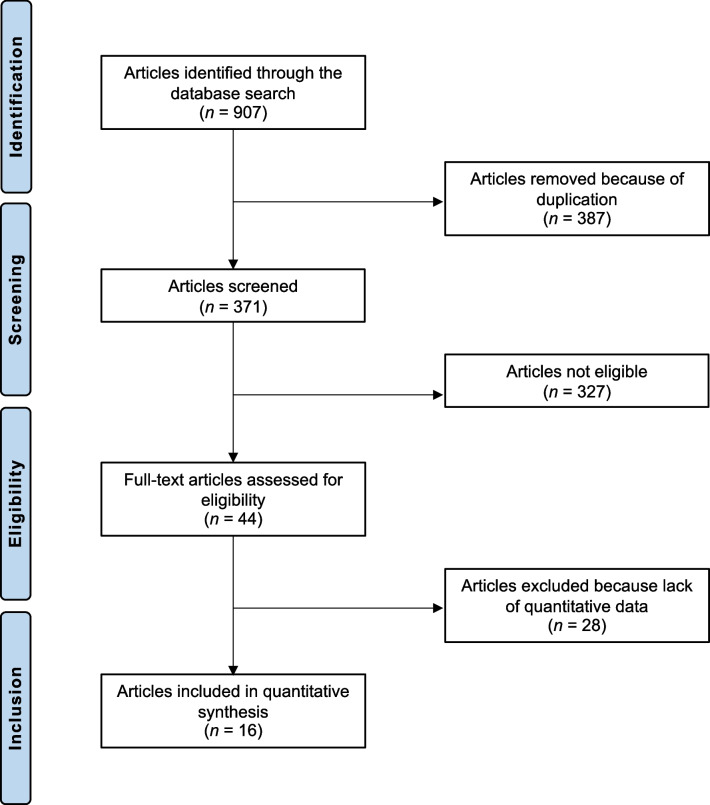
Flowchart of the literature search

A systematic review of each topic was conducted using electronic databases, including PubMed, MEDLINE, Web of Science, EMBASE, and Cochrane Library, to identify relevant studies published until 1 October 2023. The search utilized specific terms related to each topic, combined with the terms " Chronic Achilles Tendon Rupture ", “old Achilles Tendon Rupture”, “neglected Achilles Tendon Rupture”, or " Chronic Rupture of Achilles Tendon ". The inclusion criteria focused on studies that reported risk–benefit outcomes for each topic, while non-clinical studies, reviews, and study protocols were excluded. To provide a concise overview of the findings from the studies included, the researchers evaluated the design of each study and assessed the potential for bias using the Cochrane Handbook for Systematic Reviews of Interventions and the Newcastle–Ottawa scale. The latter is specifically designed to assess non-randomized studies. The results of the literature search are shown in Fig. 1.

### Determination of evidence level and grading of recommendations

The determination of recommendation grades followed the GRADE (Grading of Recommendations Assessment, Development, and Evaluation) approach [55], which categorizes evidence levels according to different study types. Well-conducted randomized controlled trials (RCTs) or high-quality systematic reviews of RCT were classified as high-quality evidence (A), while observational studies (lesser quality RCTs or strong evidence from observational studies) were regarded as having a moderate-quality evidence (B). RCTs with significant limitations, or evidence from observational studies with a high risk of bias, or cohort studies were classified as low-quality evidence (C). Case reports and expert opinions were considered to have the very low-quality evidence (D).

GRADE assigns recommendations into two categories:

- Strong recommendation: This indicates that the desirable effects of an intervention clearly outweigh the undesirable effects (or vice versa) for most patients. Most individuals would choose the recommended intervention.
- Weak recommendation: This indicates that the desirable effects of an intervention likely outweigh the undesirable effects (or vice versa) for most patients, but the balance may vary depending on individual circumstances or patient preferences.

The strength of the recommendation is based on several factors, including the quality of evidence, the balance between benefits and harms, values and preferences, costs, feasibility, and resource implications. Hence, a recommendation grade of aA signifies strong and high-quality evidence, while a grade of bD indicates weak and very low-quality evidence.

## Results and discussion

### What are the indications for surgical treatment of CATR?

*Recommendation*. In (1) patients exhibit functional impairment (i.e., pain, inability to perform a single heel rise, disability in walking and climbing stars) after 6 months of conservative management; (2) athletes or active individuals; (3) physical examination reveals positive calf squeeze and knee flexion tests; (4) MRI or ultrasound scans confirmed CATR, a surgical management is indicated. (aC).

*Statement*. No investigations related to the surgical indications for CATR or a comparison between surgical and conservative treatments for CATR were identified. However, studies focusing on the surgical management of CATR were reviewed, specifically those with evidence levels A to D. The researchers carefully analyzed the inclusion criteria of the identified studies to observe and determine the surgical indications for CATR. Following the above search strategy, 16 studies were included [33, 56–70], of which 1 study was grade A [56], 1 study was grade B [57], 6 studies were grade C [33, 58–62], and 8 studies were grade D [63–70]. The most common conditions for which surgical management is indicated were CATR patients exhibiting signs of functional limitation, such as pain or tenderness, inability to execute a single heel raise, or repetitive single-leg heel raise endurance exercises, as well as difficulty in walking and ascending stairs, along with ankle swelling; these symptoms were accompanied by positive results in the call squeeze and knee flexion [71], and their diagnoses were confirmed through MRI or ultrasound examinations (16 studies). Across all the studies, it was evident that preoperative imaging tests were routinely obtained, signifying that surgical judgments should rely on the corresponding imaging outcomes, with ultrasound and MRI being the most frequently employed supplementary examinations. In 3 studies [61, 69, 70], patients received conservative treatment for a period of 6 months before undergoing surgery, which included physical therapy and the use of anti-inflammatory medications. In the future, controlled studies comparing non-surgical and surgical treatments for CATR will be necessary to more conclusively establish surgical criteria. However, the ethics behind performing such studies involving non-surgical options are challenging, given the present evidence which suggests that surgery is associated with better outcomes [21, 23, 25, 26]. The generalities, patient characteristics, and main findings of the included studies are shown in Table 1.

**Table 1:** Generalities, patient characteristics, and main results of the included studies

References	Journal	Level of evidence	Patients (*n*)	Mean age (*y)*	Woman (*%*)	Main findings
[57]	Int Orthop	B	21	40.3	28.6	Single-incision flexor hallucis longus transfer for chronic Achilles tendon ruptures is a simple method with minimal morbidity and complications. The technique resulted in great patient satisfaction as well as excellent functional and clinical outcomes
[61]	Ann Transl Med	C	8	55.0	25.0	Autologous quadriceps tendon graft (in bone-tendon configuration) has proved as a simple technique that offers good results to patients with tissue defects in the Achilles tendon
[70]	Foot Ankle Surg	D	13	52.8	69.0	FDL tendon transfer represents an operative alternative in treating chronic Achilles tendon disorders. Good clinical outcomes with low complications and donor site morbidity were reported
[33]	BMC Musculoskelet Disord	C	24	50	29.0	Percutaneous repair of neglected Achilles rupture using the index technique proved a satisfactory patient-reported and objective measurement at a one-year follow-up. With only minor transient complications
[58]	J Clin Med	C	17	42.8	17.6	When an extensive defect is present, the reconstruction with an Achilles tendon allograft can be considered a proper treatment option, as it does not show a higher rate of complications than autologous techniques achieving similar functional outcomes
[67]	Bone Joint J	D	20	60.0	20.0	Transtendinous FHL transfer for neglected tendon Achilles ruptures, with a long harvest to allow reattachment of the triceps surae, provides reliable long-term function and good ankle plantarflexion strength. Despite the loss of strength in hallux plantar flexion, there is little comorbidity from the FHL harvest
[62]	Biomed Res Int	C	29	40.3	20.7	Surgical options can be determined by evaluating the presence of the Achilles tendon stumps and the gap length, which can avoid using the nearby tendon and yield satisfactory functional results
[60]	J Foot Ankle Surg	C	50		42	Using the modified spoon-shaped medial incision in the surgical repair of a chronic Achilles tendon rupture seems to be a safe and effective method that may reduce risk of incision skin necrosis and offers better function in patients with a chronic Achilles tendon rupture
[69]	Clin Sports Med	D	32			Peroneus brevis tendon transfer is an effective surgical technique for neglected Achilles tendon rupture. If the tendon gap is greater than 6 cm, a free gracilis autograft is indicated
[56]	Foot Ankle Surg	A	62	44.8	37.1	Free semitendinosus transfer, peroneus brevis transfer, and FHL tendon graft all resulted in significant functional improvement, and most patients were able to return to sports. No clear advantage of one technique over the others was shown
[68]	J Bone Joint Surg Am	D				Local tissue, local tendons, and allografts can be used to reconstruct the tendon, and end-to-end repair is possible if the gap is < 2.5 cm. Compared with acute injuries, chronic injuries are associated with a higher rate of postoperative infection and more prolonged recovery
[63]	Indian J Orthop	D	12	47.0	33.3	Combined surgical technique of gastrosoleus turndown flap augmentation with V–Y plasty for repairing complex defects in chronic Achilles tendon tears is a fair option with satisfactory functional outcome and fewer complications
[64]	Foot Ankle Orthop	D	10	51.7	40.0	The SLLS technique accompanied by autologous semitendinosus tendon grafting provided successful operative outcomes for patients with chronic Achilles tendon rupture regardless of the size of the defect, and thus long-term orthotic use was not needed after surgery
[66]	Foot Ankle Int	D	22	69.0	27.3	Chronic Achilles tendon ruptures were successfully treated by an all-endoscopic procedure. The endoscopically assisted FHL transfer provided excellent results while benefiting from the minimally invasive procedure advantages. However, it entailed some technical challenges and may not be suitable for less experienced surgeons
[59, 106]	Knee Surg Sports Traumatol Arthrosc	C	29	39.0	0.0	Endoscopy allowed scar tissue and adhesions to be removed, allowing the tendon ends to be mobilized out of the small proximal and distal incisions. Minimally invasive technique may result in a lower wound complication incidence and provide better early functional recovery and return to moderate-intensity exercise time than the conventional open procedure in treating chronic Achilles tendon ruptures
[65]	Orthop J Sports Med	D	19	46.7	10.5	Chronic Achilles tendon ruptures were successfully treated via minimally invasive reconstruction using a double-bundle FHL, which provided excellent functional improvement. It is best suited for patients with complex requirements who are at high risk for wound complications

*FDL* flexor digitorum longus, *FHL* flexor hallucis longus, *SLLS* side-locking loop suture

### Is it possible to primary repair the stump of the CATR?

*Recommendation*. When the defect of the two stumps of the CATR is no more than 2 cm, primary repair after refreshed the tendon edges can be attempted (aD).

*Statement*. Direct repair of the stump of the CATR in end-to-end fashion would be an ideal option. However, owing to the tendon adhesions and atrophy of the gastrocnemius, suture the stumps of the CATR makes great challenging. Although according to the Myerson’s classification [72] and Kuwada’s classification [73], a tendon defect of 2 to 3 cm could be managed with end-to-end repair, the results of these guidelines have not been assessed in a scientific fashion, even by the authors reporting them. In extreme dorsiflexion, although some defects can be directly sutured, this will increase the tension on the tendons, prolonging the postoperative recovery time and mobilization [40, 44]. When the defect is minimal, primary repair has the advantage of preserving the patient's native tissue and restoring the original anatomy of the Achilles tendon. It can potentially lead to faster healing and recovery, as well as reducing the need for more complex procedures like tendon flap or tendon grafting [74, 75]. We stress, however, that no comparative study has been performed, and that, given the atrophic nature of the tendon stumps in CATR, it does make sense to use grafts to bring new vital tissue to the chronic rupture site.

### When should tendon flaps be used?

*Recommendation*. V‐Y advancement tendon flap is a safe and reliable strategy for CATR with the gap less than 5 cm. Local fascial turndown flaps can be used for an anatomic repair of CATR with a large gap more than 5 cm. Large scar, calf atrophy and reduction of tendon strength are the major drawbacks. (aC).

*Statement*. Lin et al. [76] employed V‐Y tendon plasty on 20 patients with CATR who had a tendon gap of 5 cm (ranging from 4 to 9 cm). The study reported a significant improvement in AOFAS and ATRS scores (*P* < 0.001) after a follow-up period of 32.8 months. However, isokinetic strength analysis was not performed. Guclu et al. [77] performed a study on 17 patients with CATR who underwent V–Y tendon plasty with a fascial turndown flap following a debridement procedure for an average 6 cm Achilles tendon defect (ranging from 4.5 to 8 cm). Patients experienced a significant reduction in plantarflexion peak torque at both 30 and 120 degrees, which was attributed to gastrocnemius recession and correlated with the size of the tendon defect. Additionally, the mean calf atrophy measured 3.4 cm (with a range of 1–6 cm) after an average follow-up period of 195 months. Raju et al. [63] presented a study on 12 CATR patients with a tendon gap exceeding 8 cm, where they utilized a gastrosoleus turndown flap along with V–Y plasty. After an average follow-up period of 34 months, the major limitations observed were a preoperative calf diameter loss of 3.4 cm and a noticeable decline in plantar flexion strength on the affected side. Studies involving larger sample sizes and a prospective study design are desirable [78].

### When should tendon Transfer be used?

*Recommendation*. When the defect is between 3 and 6 cm, a local tendon transfer [peroneus brevis tendon transfer or flexor hallucis longus (FHL) transfer] procedure should be taken into consideration. (aC).

*Statement*. Peroneus brevis tendon transfer and FHL tendon transfer can effectively reconstruct the Achilles tendon defect less than 6 cm without considerable tension [79]. They reduce the necessity for extensive soft tissue procedures such as turn down flaps or V–Y plasty [80, 81]. Biomechanically, the peroneus brevis tendon exhibits increased load to failure compared to the tendon of FHL. The current evidence suggests that individuals who undergo peroneus brevis tendon transfer experience a more gradual return to sports in comparison with those who undergo FHL transfer [56, 82, 83]. It should be noted that peroneus brevis tendon transfer results in most patients regaining their ability to engage in preinjury sports and daily activities [84]. The operated ankle may have lower peak torque and eversion strength compared to the unaffected limb, although such difference, though statistically significant, are of dubious clinical significance [85]. Maffulli et al. [86] employed peroneus brevis tendon transfer to reconstruct the CATR with the defect of 4.0 to 6.5 cm in 16 patients. At the 15.5-year review, the patients retained good functional outcomes. However, permanently impaired ankle plantar flexion strength and decreased calf circumference are the major complications. Peroneus brevis tendon transfer is viable to enable recreational athletes and non-athletes to resume their sports activities, but it may not be the appropriate solution for young competitive athletes [87, 88]. Patients who underwent FHL transfer experienced significant satisfaction without any impact on the function of their hallux [57, 89–91]. On the other hand, patients who underwent open FHL transfer retained nearly normal maximum strength but exhibited reduced endurance compared to the unaffected limb [92–96]. Additional augmentation following FHL transfer did not show statistically significant results [97, 98]. Further augmentation following FHL transfer did not produce any further positive effects and is not warranted.

### Peroneus Brevis Tendon transfer versus FHL transfer-which is better?

*Recommendation*. Patients undergoing either procedure could receive comparable long-term functional results (aC).

*Statement*. No study comparing the two techniques was found to provide conclusive evidence of one method having a clear advantage over the other. Maffulli et al. [56] conducted a comparison between peroneus brevis tendon transfer (*n* = 20) and FHL transfer (*n* = 21) in CATR patients with a gap of less than 6 cm. Patients who underwent peroneus brevis tendon transfer had a delayed return to sports but a higher likelihood of resuming high impact sports when compared to those who received the FHL transfer. According to Maffulli et al. [79, 86, 99] and other researchers [84, 100–104] there was a reported improvement in Achilles tendon total rupture score and American Orthopedic Foot and Ankle Society Score (AOFAS) following peroneus brevis tendon transfer. An additional 20 studies [97, 105–123] reported comparable outcomes regarding the Achilles tendon total rupture score and AOFAS after performing the open FHL Transfer. Patients undergoing either peroneus brevis tendon transfer or FHL transfer can anticipate experiencing similar functional outcomes.

No significant functional deficit has been documented, and although a statistically significant loss in peroneus brevis strength has been reported, patients do not report any increase in episode of lateral ankle instability [124]. The use of peroneus brevis tendon transfer is therefore to be considered a suitable tendon transfer option in selected patients.

### Open versus endoscopic FHL transfer procedure for CATR-which is better?

*Recommendation*. Patients undergoing either procedure could receive comparable long-term functional results (bC).

*Statement*. Currently, there is no available comparative study examining the differences between open and arthroscopic FHL transfer for CATR.

The use of flexor hallucis longus (FHL) tendon transfer presents a suitable option to restore continuity in CATR patients with excellent results [105–115, 122, 125–129]. Notably, certain researchers have recently presented endoscopic approaches for the transfer of FHL. A total of 22 patients with CATR underwent endoscopic FHL Transfer, and the average follow-up period was 30.5 months [66]. The AOFAS score improved from 55 before the operation (with a range of 26–75) to 91 (with a range of 74–100) postoperatively. Additionally, all patients successfully resumed their daily activities without any complications or difficulties. A systematic review [130] found that endoscopic FHL transfer resulted in reduced complications compared to open FHL reconstruction, while still achieving favorable clinical outcomes and allowing patients to return to their preinjury activity levels. Despite the appealing advantages of this technique as a minimally invasive procedure, the surgeon remains vigilant about the potential complications [129]. In the study by Zou et al. [65], 19 patients with CATR underwent endoscopic FHL transfer, and they were followed up for an average of 31 months. The Achilles Tendon Total Rupture Score and AOFAS scores showed significant improvement, increasing from 23.3 ± 10.3 and 52.1 ± 12.4 to 98.3 ± 9.2 and 97.5 ± 18.9, respectively. Notably, 12 relatively young patients were able to achieve a return to their preinjury activity levels. Nevertheless, given the lack of comparability in sample sizes between the groups, further studies are required to reach a definitive conclusion [131].

### When should free tendon graft be used?

*Recommendation*. Free tendon graft would be recommended if the gap of the CATR is larger than 6 cm (aC).

*Statement*. While the FHL transfer and gastrosoleus turndown flap techniques have been employed in patients with CATR involving gaps exceeding 6 cm, the utilization of free tendon grafts was more prevalent [37, 69, 132–141]. In CATR patients in whom the gap between the stumps was greater than 6 cm, Maffulli et al. [142, 143] employed free grafts from the gracilis tendon for the reconstruction procedure. In the cohort of 15 patients, an average follow-up of 10.9 years revealed sustained positive functional outcomes, despite enduring limitations in ankle plantar flexion strength and reduced calf circumference [142]. Performing minimally invasive reconstruction of the Achilles tendon in patients with a gap exceeding 6 cm, utilizing a free graft from the ipsilateral semitendinosus tendon, leads to notable improvement of symptoms and enhancement function [144–149]. However, patients should be advised that full recovery of calf circumference and ankle plantarflexion strength is unlikely to be achieved [145]. Nilsson et al. [150] conducted endoscopic reconstruction using ipsilateral semitendinosus autografts in the management of CATR, with favorable results in a cohort of 22 patients. In a series of 15 CATR patients who underwent endoscopic-assisted reconstruction using autografts from the hamstring muscles [151], isokinetic testing at a two-year follow-up revealed a minor and statistically not significant difference in strength between the affected and unaffected sides. While the use of tendon allograft to patients with CATR has yielded favorable clinical outcomes, their effectiveness necessitates further validation and confirmation [152–158].

### When to start range of motion and weight bearing?

*Recommendation*. Early range of motion of the knee and toes is advised to avoid the limitations of joint immobility. Patient can be encouraged to bear as much weight as possible on the second day after surgery aided by below-the-knee cast and elbow crutches. Two weeks post-surgery, patients can be granted clearance for full weight bearing while keeping the Aircast boot in place. (aC).

*Statement*. No study specifically addresses the issue of postoperative care after surgical treatment of CATR. In general, early functional treatment yielded superior subjective outcomes while displaying no increased re-rupture rates compared to postoperative immobilization [40, 159, 160]. Immediate loading and ankle motion led to improved overall health and vitality at six month postoperatively [161]. Patients were advised to engage in active knee and toe flexion and extension, along with isometric calf muscle exercises and straight-leg raises [162, 163]. Early weight bearing and immediate functional rehabilitation following surgical treatment for CATR appears to improve long-term functional results, including the ability to resume work [164–168]. A below-knee cast was prescribed for the first 2 weeks following the surgery, and patients were allowed to bear weight on the metatarsal heads during this period if they could tolerate it [79, 99, 124, 139, 143–145, 148]. The Aircast walking boot could be employed to support early full weight bearing for the following six weeks [169]. Subsequently, the boot was taken off, and patients were allowed to walk with full weight bearing after 8 weeks.

### When to return to sports?

*Recommendation*. Resuming regular sports activities should not occur earlier than 12 weeks post-surgery, and engaging in strenuous sports should be postponed for a minimum of 12 months. (aC).

*Statement*. A recent meta-analysis, drawing data from 15 studies, revealed that the rate of return to play stood at 76% among professional athletes, with an average time to return to sports of 11 months [170]. These findings align with the return to sports rate of 80% reported in Zellers and colleagues' comprehensive systematic review and meta-analysis, which encompassed 85 studies [171]. Usuelli et al. [172] studied 8 patients with CATR who underwent a semitendinosus tendon graft transfer. Six of these 8 patients successfully resumed their preinjury sports activities, achieving this milestone at an average of 7.0 months. The rate of athletes returning to sport after experiencing a CATR still stands at 80%. However, it remains challenging for them to reach their baseline performance levels until 2 to 3 years following surgery, which can have a significant impact on an athlete's overall career [173–177].

## Conclusion

This guideline outlines the prevailing surgical approaches for CATR presently. It aims to offer surgeons valuable insights to inform their surgical decisions for CATR patients based on the available evidence. Additionally, we expect that this guideline will offer valuable insights for orthopedic researchers as they formulate future study methodologies, taking into account the current understanding of surgical treatment for CATR.

The main *recommendation*s are outlined as follows:

1. Surgery is recommended for patients who still experience CATR symptoms despite unsuccessful conservative treatment and show signs of CATR on both physical and imaging evaluations.
2. Primary repair procedures are recommended in CATR patients when the defect is no more than 2 cm
3. V‐Y advancement tendon flap is recommended for CATR patients with the gap less than 5 cm; Fascial turndown flap is available for the patients with CATR when the gap more than 5 cm. However, these techniques produce large wounds and have a respective impact on the biomechanics of the gastrocnemius-Achilles tendon complex.
4. Tendon transfer procedures, such as the Peroneus Brevis Tendon Transfer and FHL Transfer, are advisable to address gaps ranging from 3 to 6 cm in patients with CATR.
5. Both open and endoscopic FHL transfer techniques are suitable for CATR patients undergoing tendon transfer procedures.
6. Free tendon graft procedures are recommended for CATR patients with the gap more than 6 cm.
7. Early range of motion of the knee and toes, along with weight-bearing support from a below-the-knee cast and elbow crutches starting on the second day after CATR surgery are recommended; full-weight bearing in suitable orthosis is recommended 2 weeks postoperatively.
8. For CATR patients, return to regular sports would be recommended 12 weeks post-surgery, and return to strenuous sports is recommended 12 months after the surgery.

## Data Availability

Data are available from the co-corresponding authors following reasonable requests.
